# Epidemiologic profile of hepatitis C virus infection and genotype distribution in Burkina Faso: a systematic review with meta-analysis

**DOI:** 10.1186/s12879-021-06817-x

**Published:** 2021-11-01

**Authors:** Serge Ouoba, Jean Claude Romaric Pingdwinde Ouedraogo, Moussa Lingani, Bunthen E, Md Razeen Ashraf Hussain, Ko Ko, Shintaro Nagashima, Aya Sugiyama, Tomoyuki Akita, Halidou Tinto, Junko Tanaka

**Affiliations:** 1 Department of Epidemiology, Infectious Disease Control and Prevention, Graduate School of Biomedical and Health Sciences, Hiroshima University, 1-2-3, Kasumi, Minami-ku, Hiroshima, 734-8551 Japan; 2 Unité de Recherche Clinique de Nanoro (URCN), Institut de Recherche en Science de la Santé (IRSS), Nanoro, Burkina Faso; 3 Département de Médecine et Pharmacopée Traditionnelles, Pharmacie, Institut de Recherche en Sciences de la Santé (IRSS), Ouagadougou, Burkina Faso; 4 Payment Certification Agency (PCA), Ministry of Health, Phnom Penh, Cambodia

**Keywords:** Hepatitis C, Seroprevalence, Prevalence, Genotype, Systematic review, Burkina Faso

## Abstract

**Background:**

Detailed characteristics of Hepatitis C virus (HCV) infection in Burkina Faso are scarce. The main aim of this study was to assess HCV seroprevalence in various settings and populations at risk in Burkina Faso between 1990 and 2020. Secondary objectives included the prevalence of HCV Ribonucleic acid (RNA) and the distribution of HCV genotypes.

**Methods:**

A systematic database search, supplemented by a manual search, was conducted in PubMed, Web of Science, Scopus, and African Index Medicus. Studies reporting HCV seroprevalence data in low and high-risk populations in Burkina Faso were included, and a random-effects meta-analysis was applied. Risk of bias was assessed using the Joanna Briggs institute checklist.

**Results:**

Low-risk populations were examined in 31 studies involving a total of 168,151 subjects, of whom 8330 were positive for HCV antibodies. Six studies included a total of 1484 high-risk persons, and 96 had antibodies to HCV. The pooled seroprevalence in low-risk populations was 3.72% (95% CI: 3.20–4.28) and 4.75% (95% CI: 1.79–8.94) in high-risk groups. A non-significant decreasing trend was observed over the study period. Seven studies tested HCV RNA in a total of 4759 individuals at low risk for HCV infection, and 81 were positive. The meta-analysis of HCV RNA yielded a pooled prevalence of 1.65% (95% CI: 0.74–2.89%) in low-risk populations, which is assumed to be indicative of HCV prevalence in the general population of Burkina Faso and suggests that about 301,174 people are active HCV carriers in the country. Genotypes 2 and 1 were the most frequent, with 60.3% and 25.0%, respectively.

**Conclusions:**

HCV seroprevalence is intermediate in Burkina Faso and indicates the need to implement effective control strategies. There is a paucity of data at the national level and for rural and high-risk populations. General population screening and linkage to care are recommended, with special attention to rural and high-risk populations.

**Supplementary Information:**

The online version contains supplementary material available at 10.1186/s12879-021-06817-x.

## Background

Hepatitis C virus (HCV) infection is a bloodborne disease which is globally distributed that mainly affects developing countries. In 2019, an estimated 58 million chronic cases were reported in the world [1], 75% of which occurred in low- and middle-income countries (LMIC) [2]. Worldwide, new HCV infections occur in high-risk populations, including people who inject drugs (PWID) and men who have sex with men (MSM) [3]. In sub-Saharan Africa, HCV is mainly transmitted via unsafe medical practices and contaminated blood transfusion [4]. Other transmission routes include needlestick injury in healthcare workers, mother-to-child transmission (MTCT), and social practices such as piercing and tattooing [3].

HCV infections are usually asymptomatic and silently progress over time. Up to 25% of new infections spontaneously resolve [5], and the remaining evolve to chronicity with complications such as liver cirrhosis and hepatocellular carcinoma (HCC) [3]. Since no vaccine is available to prevent HCV infection and its chronic consequences, screening and treatment of cases using effective direct-acting antivirals (DAA) are required [6]. HCV antibody (anti-HCV) assays are typically positive within 4–10 weeks after the initial infection, persist lifelong, and indicate a current or past exposure to the virus [7]. Thus, the WHO requires a confirmatory test by detecting HCV ribonucleic acid (HCV RNA) or core antigen (HCVcAg) [8].

In Burkina Faso, a West African country, the epidemiologic patterns of HCV infection are poorly documented. The only nationwide study conducted in 2010 reported a seroprevalence of 3.6% (95% CI: 3.3–3.8) in the general population [9]. In addition, various seroprevalence data classify the country among those of intermediate or high seroprevalence (seroprevalence ≥ 2% or ≥ 5%, respectively) [10–12]. However, detailed characteristics of the infection in specific populations and geographic areas are scarce, representing a limitation for implementing effective control strategies. Thus, this study aimed to provide detailed knowledge on the epidemiologic profile of HCV infection in Burkina Faso by synthesizing data on HCV seroprevalence in various settings and populations at-risk in Burkina Faso. Secondary objectives included the prevalence of HCV RNA and the distribution of genotypes in the country.

## Methods

### Study design and guidelines

We conducted a systematic review with meta-analysis following the Joanna Briggs Institute guidelines for systematic reviews of studies reporting prevalence data [13]. This manuscript is reported according to the Meta-analysis Of Observational Studies in Epidemiology (MOOSE) recommendations [14].

### Study setting

Burkina Faso is a landlocked country in West Africa with an overall population of 20,487,979 people in 2019 [15]. The country is divided into 13 regions, with the central region accounting for 14.8% of the total population. About one in four inhabitants live in rural areas. This developing country is characterized by the persistence of tropical infections and the epidemiological transition to non-transmissible diseases.

### Data source and study selection

We searched the following databases to identify records published between 1990 and 2020: PubMed, Web of Science, Scopus, and African Index Medicus. A complete list of the search queries is shown in Additional file 1: Table S1. Besides, a manual search was conducted on African Journals Online (AJOL), Google Scholar, the reference lists of eligible reports, and the electronic library of the University Joseph Ki-Zerbo (www.biblio-ujkz.com), the largest university in the country. The last search was conducted on July 1, 2020, and reports in English or French were eligible. After removing duplicates using the EndNote reference manager, two independent investigators (SO and JCRPO) screened the records based on their titles and abstracts. Those deemed relevant were retained for full-text review. Any report (journal article, conference abstract, government report) of HCV antibody testing among people living in Burkina Faso was considered for inclusion. Reports that did not mention sample size and number of cases (or prevalence) were excluded. Disagreements between the two investigators were solved by discussion. When there was no consensus, the final decision was made by a third investigator (ML).

### Data extraction

A pre-piloted standardized electronic data extraction form was used to extract relevant data: authors names, year of publication, study setting, study design, sampling method, study period, characteristics of study subjects (age, sex, location, co-morbidities), sample size, number of participants positive for anti-HCV, and type of biological assay. We also extracted HCV RNA and genotyping data when available. In this paper, a “report” refers to any document mentioning HCV seroprevalence data, and a “study” relates to the measurement of anti-HCV antibodies in a given population or setting (rural/urban). Therefore, one report may describe multiple studies, and in this case, we extracted data for each study separately. When data were suspected to originate from the same source, only one publication was included.

### Quality appraisal

Joanna Briggs Institute checklist for prevalence studies was used to appraise the methodological quality of the studies [13]. Before independently using the tool, the reviewers agreed on the minimum acceptable information for each item. Sample size was considered adequate when more than 411 subjects were included. This was based on an assumed seroprevalence of 3.6% [9], a precision of 1.8%, and a confidence level of 95%. For the sampling procedure, only probabilistic sampling was considered representative of the target population. Anti-HCV seroprevalence data was considered valid when the diagnostic was based on biological testing.

### Data synthesis and analysis

We categorized the study populations into two groups based on the risk of HCV transmission. The low-risk group included the general population, blood donors, pregnant women, and children. People living with Human Immunodeficiency Virus (HIV), sex workers, MSM, and healthcare workers represented the high-risk group.

A meta-analysis of proportion was performed using the 'meta' and 'metafor' packages in the statistical program R. Data were transformed using the Freeman-Tukey double arcsine method to account for small proportions. The Dersimonian and Laird method, based on the random-effects model, was used to perform the meta-analysis and summarize data in a forest plot. Confidence-interval (CI) for individual studies proportions were calculated using the Clopper-Pearson method. Heterogeneity was assessed based on the Cochran Q test and quantified by the I^2^ index. Heterogeneity was considered significant when the p-value of the Cochran Q test was less than 0.05. The level of heterogeneity was rated as high, medium, or low when the I^2^ index was 75%, 50%, and 25%, respectively [16]. Publication bias was evaluated graphically by a funnel plot of the transformed proportion against the sample size [17]. We used the Egger test to assess the symmetry of the plot (p < 0.1).

## Results

### Search results

**Fig. 1:**
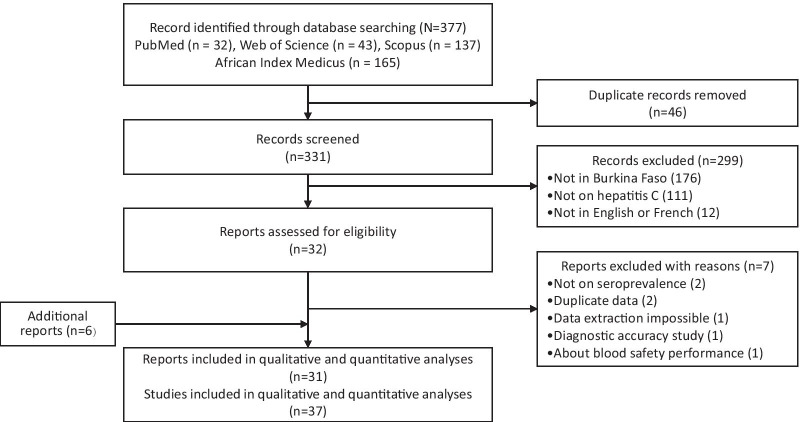
Flowchart of records identification and study selection. A “report” refers to any document mentioning HCV seroprevalence data, and a “study” relates to the measurement of anti-HCV antibodies in a specific population or setting. Of the 31 reports that met the review criteria, four reported seroprevalence data on separate study subjects or settings (as shown in Table 1), and each seroprevalence data was extracted as an individual study. Therefore, 31 reports met the criteria for this review, and 37 prevalence data, referred to as “studies”, were extracted

A total of 377 records were identified from the database search. After duplicates exclusion and titles and abstracts screening, 32 reports were selected for full-text review. Of these, 25 met the inclusion criteria, and six reports were further included, resulting in 31 reports. Finally, the qualitative review and the meta-analysis included 37 studies covering a population of 169,635, as some reports provided data on multiple populations or settings. The selection process is summarized in Fig. **1**.

### Study characteristics

The details about the characteristics of each study are reported in Table 1. Studies were conducted between 1994 and 2018. The central region was the most represented (22/37 studies), and seven studies addressed the rural area. The cross-sectional design (33/37) was the main method used, with only eight studies (21.62%) using a probabilistic sampling. Sample size was considered adequate (> 411) in 46.0% of the studies, and the presence of anti-HCV antibodies was ascertained by biological assays in 34 studies (92.0%) (Additional file 1: Table S2). Enzyme immunoassays (ELISA, CLEIA) were the most used (24/34 studies), followed by rapid tests (7/34) and immunoblotting (3/34).

**Table 1:** Characteristics of included studies reporting HCV seroprevalence in Burkina Faso between 1990 and 2020

First author, Year of publication	Study design	Sampling method	Data collection period	Setting	Region	Study subjects	Mean age or range (in years)	Sample size	Number of anti-HCV positive	Number of HCV RNA positive	Assay for Anti-HCV testing
Jeannel, 1998a [18]*	CS	Random	1994–1995	Urban	Haut Bassins	General population	23.1	638	20	3	Immunoblot
Jeannel, 1998b [18]*	CS	Consecutive	1994–1995	Rural	Haut Bassins	General population	37	327	27	23	Immunoblot
Ilboudo, 2003 [35]	CS	NR	2000	Urban	Center	Pregnant women	25.1 ± 5.5	288	12		ELISA/EIA
Simpore, 2005 [21]	CS	Random	2001–2002	Urban	Center	Pregnant women	25.9 ± 5.8	547	18	5	Immunoblot
Serme, 2006 [19]	CS	NR	2002	Urban	Center	Pregnant women	27.2 ± 6.4	200	17	4	ELISA/EIA
Kania, 2009 [36]	CS	Random	2002	Urban	Center	Blood donors	18–65	500	26		ELISA/EIA
Collenberg, 2006a [37]*	CS	NR	2003	Urban	Center	Pregnant women	25.7	292	6		ELISA/EIA
Collenberg, 2006b [37]*	CS	Random	2003–2004	Rural	Boucle du Mouhoun	Pregnant women	25.3	200	5		ELISA/EIA
Collenberg, 2006c [37]*	CS	NR	2003–2004	Rural	Boucle du Mouhoun	Blood donors	25.5	89	1		ELISA/EIA
Collenberg, 2006d [37]*	CS	NR	2004	Urban	Center	Blood donors	26	102	0		ELISA/EIA
Simpore, 2006 [38]	CS	NR	2004–2005	Urban	Center	Pregnant women	25.9 ± 6.8	336	18		Rapid test
Ouedraogo A, 2012 [39]	CS	NR	2007	Urban	Center	Blood donors	28.8 ± 8.9	115	3		NR
Nagalo, 2011 [24]	CS	NR	2009	Urban	Center-West	Blood donors	24	4520	393		ELISA/EIA
Nagalo, 2012 [40]	CS	NR	2009	Urban	Center, Hauts Bassins, East	Blood donors	17–67	31,405	1964		ELISA/EIA
Zeba, 2011 [41]	CS	NR	2009	Urban	Center	Pregnant women	28.3 ± 5.6	607	13	13	Rapid test
Kirakoya-Samadoulougou, 2014 [42]	CS	NR	2010	Urban	Center, Hauts Bassins, East, Center-West	Blood donors	17–65	37,647	1996		ELISA/EIA
Meda, 2018a [9]*	CS	Random	2010–2011	Urban	National	General population	15–59	4697	101		ELISA/EIA
Meda, 2018b [9]*	CS	Random	2010–2011	Rural	National	General population	15–59	10,189	464		ELISA/EIA
Kania, 2013 [43]	CS	Consecutive	2011	Urban	Hauts Bassins	General population	29.8 ± 11.0	218	5	32	ELISA/EIA
Zeba, 2014 [44]	CS	NR	2011	Urban	Center	Blood donors	17–65	2200	97		ELISA/EIA
Tao, 2013 [45]	CS	NR	2012	Urban	Center	Blood donors	17–65	551	18		ELISA/EIA
Zeba, 2012 [20]	CS	NR	2012	Urban	Center	General population	33.2 ± 11.3	462	18		ELISA/EIA
Kissou, 2017 [46]	CS	NR	2014	Urban	Hauts Bassins	Children	7.9	70	2		ELISA/EIA
Yooda, 2019 [47]	RC	NR	2015–2017	Urban	Center	Blood donors	27.3 ± 8.8	68,391	3011		ELISA/EIA
Diarra, 2017 [48]	CS	Convenience	2016	Urban	Center	General population	31.4 ± 15.7	217	2		Rapid test
Yooda, 2018 [49]	CS	NR	2017	Urban	Center	Blood donors	27.3 ± 8.8	989	27		CLEIA
Lingani, 2020a [22]*	CS	Random	2018	Rural	Center-West	Children	2–11	240	5		CLEIA
Lingani, 2020b [22]*	CS	Random	2018	Rural	Center-West	Women	33.2 ± 7.8	240	13	1	CLEIA
Tao, 2014 [50]	CS	Convenience	NR	Urban	Center	General population	41.5 ± 12.6	995	10		Rapid test
Simpore, 2004 [51]	CS	NR	NR	Urban	Center	Pregnant women	18–44	429	26		Rapid test
Ouedraogo A, 2018 [52]	CS	Convenience	NR	Rural	Cascades, Center-South	General population	30.5 ± 10.0	450	12		Rapid test
Pietra, 2008 [53]	CS	Convenience	2008	Rural	Center-West	Healthcare workers	34.2	157	1		Rapid test
Ekouevi, 2018 [54]	CS	Consecutive	2012	NR	NR	HIV positive	NR	232	18	15	ELISA/EIA
Sawadogo, 2015 [55]	CS	NR	2012	Urban	Haut Bassins	Healthcare workers	43.6 ± 8.2	285	7		NR
Ouedraogo HG, 2018 [56]	CS	Convenience	2013	Urban	Center	MSM	22.9 ± 4.0	329	36		ELISA/EIA
Ouedraogo HG, 2019 [57]	CS	Convenience	2013	Urban	Center	Sex workers	24.9 ± 6.4	325	32		ELISA/EIA
Dah, 2019 [58]	PC	Convenience	2015–2018	Urban	Center	MSM	23.8	156	2		NR

*CS* cross-sectional, *RC* retrospective cohort, *PC* prospective cohort, *NR* not reported, *CLEIA* chemiluminescence enzyme immunoassay, *ELISA* enzyme-linked immunosorbent assay, *MSM* men having sex with men

^*^Studies issued from the same report

### Seroprevalence in the low-risk group

**Fig. 2:**
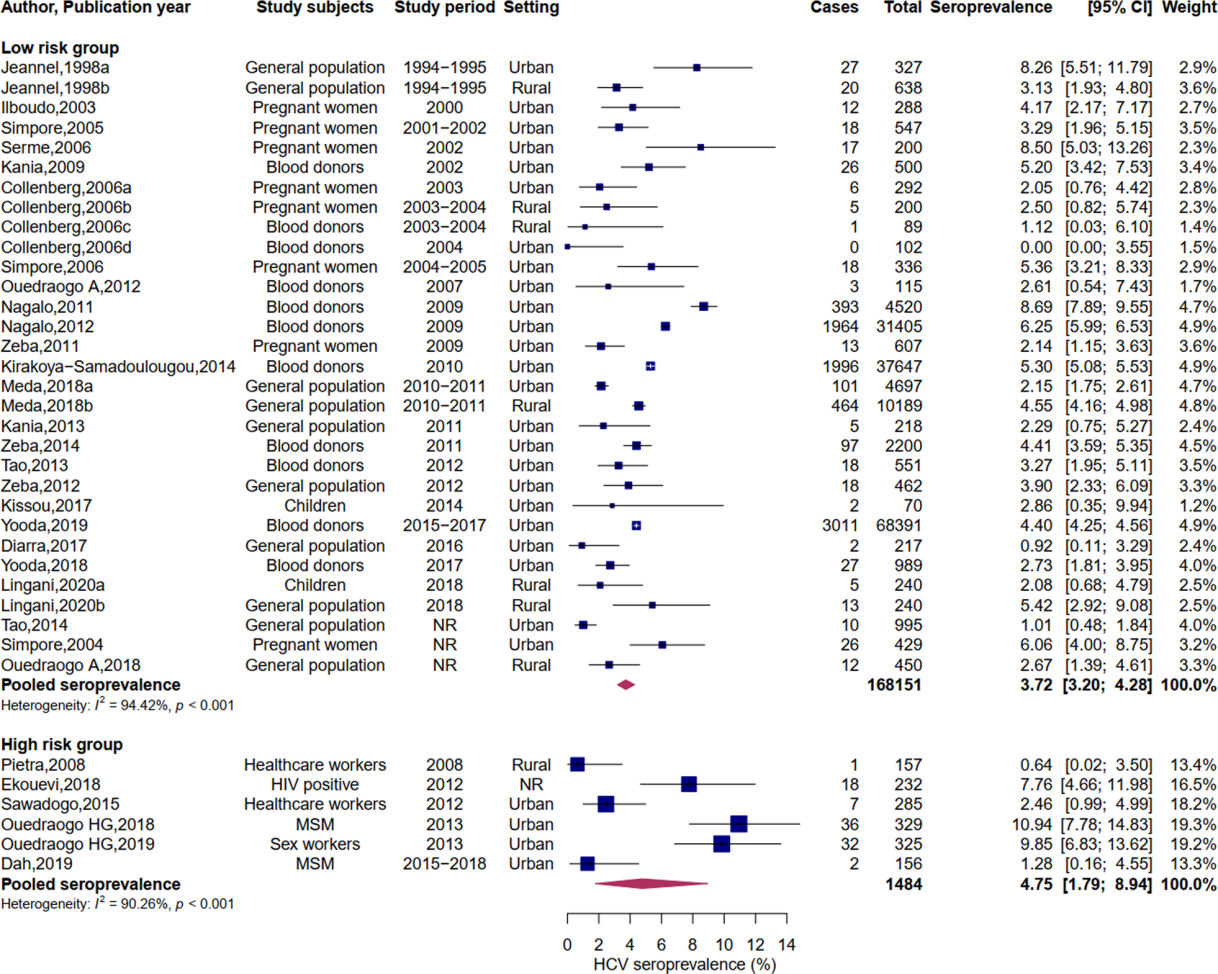
Meta-analysis of HCV seroprevalence in Burkina Faso by risk group. Pooled seroprevalence based on random-effects model. Low-risk group includes the general population, blood donors, pregnant women, and children. High-risk group is constituted of people living with Human Immunodeficiency Virus (HIV), sex workers, men having sex with men (MSM) and healthcare workers

Low-risk populations were addressed in 31 studies and involved a total of 168,151 subjects, of whom 8330 were positive for HCV antibodies. Ten studies focused on the general population, eleven on blood donors, eight on pregnant women, and two on children. The seroprevalence ranged between 0 and 8.69%, and the pooled seroprevalence was 3.72% (95% CI: 3.20–4.28). However, significant heterogeneity was found in this group (I^2^ = 94.42%, p < 0.001) (Fig. 2).

### Seroprevalence in the high-risk group

Subjects in the high-risk group were investigated in six studies: two on healthcare professionals, two on men having sex with men (MSM), one on people living with HIV, and one on sex workers. A total of 1,484 high-risk persons were studied, and 96 had antibodies to HCV. Seroprevalence varied from 1.28% to 10.94% (Fig. 2), and the combined seroprevalence was 4.75% (95% CI: 1.79–8.94). Heterogeneity was also significant in this group (I^2^ = 90.26%, p < 0.001).

### Seroprevalence by study subjects

Among populations with a low risk of infection, blood donors had the highest seroprevalence (4.51%, 95% CI: 3.73–5.35, I^2^ = 96.51%, p < 0.001), followed by pregnant women (3.95%, 95% CI: 2.67–5.46, I^2^ = 71.86%, p < 0.001). The seroprevalence in the general population was 3.11% (95% CI: 2.05–4.38, I^2^ = 92.14%, p < 0.001), and 2.11% (95% CI: 0.68–4.16, I^2^ = 0%, p = 0.59) in children. HCV seroprevalence was highest among sex workers (9.85%, 95% CI: 6.82–13.34) and HIV patients (7.76%, 95% CI: 4.63–11.59). Healthcare workers had the lowest seroprevalence (1.57%, 95% CI: 0.28–3.68, p = 0.18). The summary of the seroprevalence by study subjects is shown in Table 2.

**Table 2:** HCV seroprevalence in subgroups

Categories	Number of studies	Total population	Pooled seroprevalence	95% CI	I^2^ index (%)	Cochran Q test p-value
*Study subjects*						
General population	10	18,433	3.11	2.05–4.38	92.14	< 0.001
Blood donors	11	146,509	4.51	3.73–5.35	96.51	< 0.001
Pregnant women	8	2899	3.95	2.67–5.46	71.86	< 0.001
Children	2	310	2.11	0.68–4.16	0	0.59
Healthcare workers	2	442	1.57	0.28–3.68	44.09	0.18
MSM	2	485	5.19	0.00–18.25	94.81	< 0.001
Sex workers	1	325	9.85	6.82–13.34	NA	NA
HIV patients	1	232	7.76	4.63–11.59	NA	NA
Study setting*						
Urban	25	156,420	4.02	3.40–4.68	95.44	< 0.001
Rural	7	12,046	3.27	2.35–4.33	59.24	0.02
Decade of data collection*						
1990–2000	2	965	5.33	1.42–11.45	91.06	< 0.001
2000–2010	13	39,201	3.95	2.85–5.21	90.37	< 0.001
2010–2020	12	126,111	3.77	3.25–4.32	92.71	< 0.001

^*^Analyses performed considering only low-risk populations (general population, blood donors, pregnant women, children)

### Seroprevalence by study setting

The analysis of the seroprevalence by study setting was performed considering only low-risk groups (Table 2). Seven studies were conducted in rural areas and covered a population of 12,046 people. The pooled seroprevalence was 3.27% (95% CI: 2.35–4.33, I^2^ = 59.24%, p = 0.02). Urban populations were addressed in 24 studies and yielded a combined seroprevalence of 4.02% (95% CI: 3.40–4.68, I^2^ = 95.44%, p < 0.001).

### Seroprevalence by the decade of data collection

Only low-risk populations were considered for the analysis of HCV seroprevalence by the period of data collection. Two studies were conducted between 1990 and 2000, resulting in a pooled seroprevalence of 5.33% (95% CI: 1.42–11.45, I^2^ = 91.06%, p < 0.001). The seroprevalence decreased to 3.95% (95% CI: 2.85–5.21, I^2^ = 90.37%, p < 0.001, 13 studies) during 2000–2010 and to 3.78% (95% CI: 3.19–4.41, I^2^ = 92.71%, p < 0.001, 13 studies) between 2010 and 2020 (Table 2). However, this decrease was not significant (p for difference = 0.78).

### HCV RNA prevalence

**Fig. 3:**
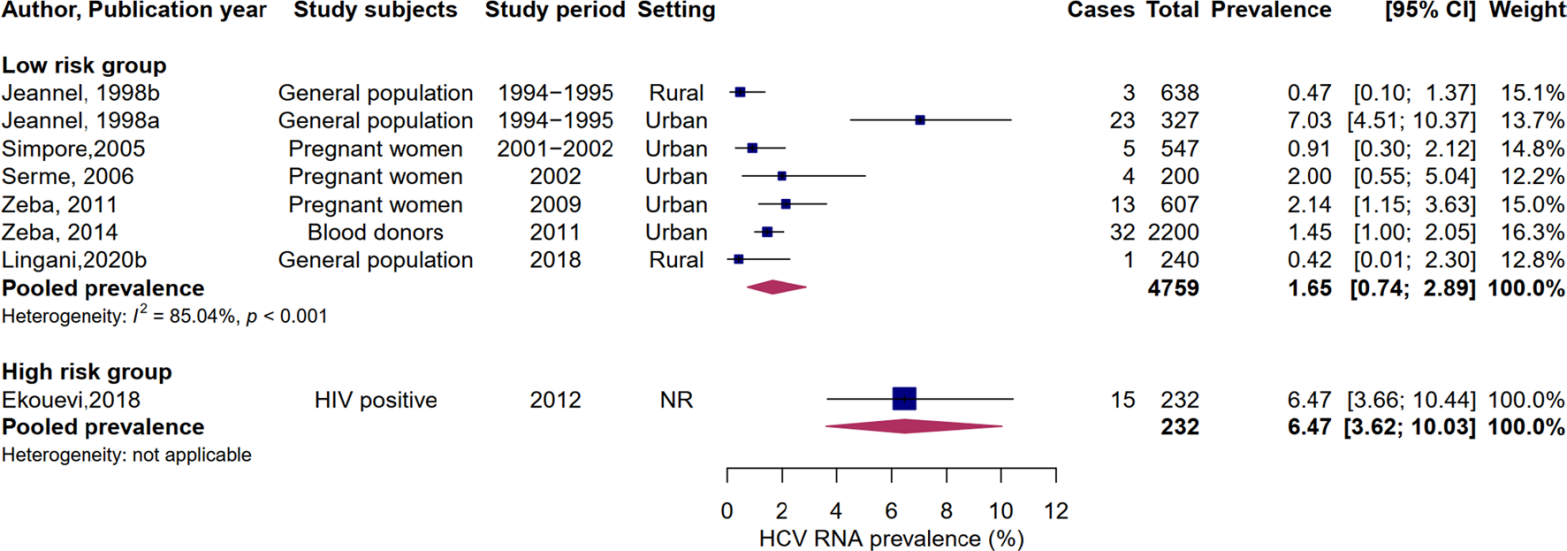
Meta-analysis of HCV RNA prevalence in Burkina Faso by risk group. Pooled prevalence based on random-effects model. Low-risk group includes the general population, blood donors, and pregnant women. Only one study evaluated HCV RNA in high-risk groups (people living with HIV)

Eight studies assessed the presence of HCV RNA, including two in rural settings and only one in the high-risk group. HCV RNA prevalence ranged from 0.47 to 7.03%. The highest prevalence estimates were found in HIV-positive patients (6.47%) and urban populations surveyed in 1994–1995 (7.03%). The seven studies that tested HCV RNA in low-risk populations included a total of 4,759 individuals, of whom 81 were positive. The meta-analysis yielded a pooled HCV RNA prevalence of 1.65% (95% CI: 0.74–2.89, I^2^ = 85.04%, p < 0.001) in the low-risk group. Figure 3 shows the forest plot of HCV RNA prevalence.

### HCV genotypes

**Fig. 4:**
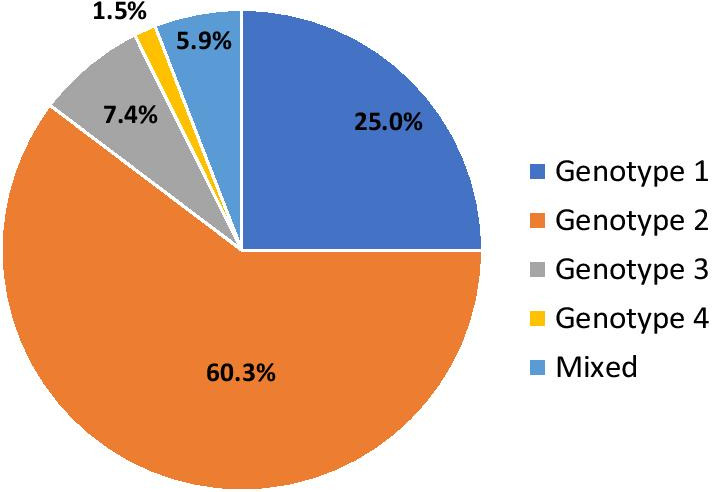
Distribution of HCV genotypes in Burkina Faso. Mixed infections consisted of genotypes 2/3 and 2/4

The genotyping of the virus was performed in six studies [18–22], and genotype 2 was the most predominant (60.3%), followed by genotype 1 (25.0%) and genotype 3 (7.4%). Mixed infections accounted for 5.9%, and genotype 4 was found in only one subject (1.5%) (Fig. 4).

### Publication bias

**Fig. 5:**
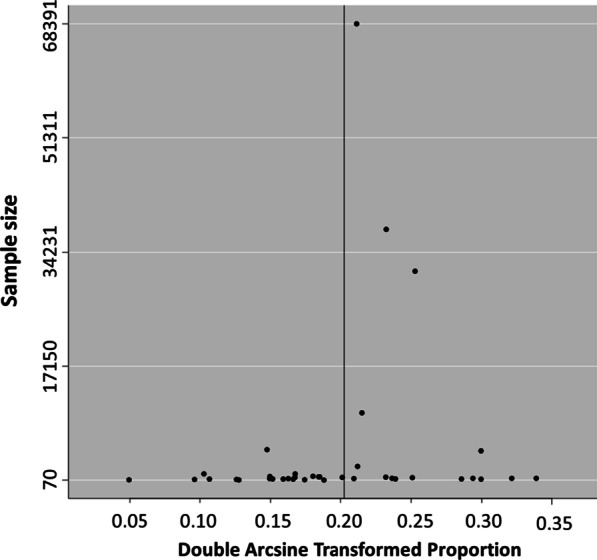
Funnel plot of HCV seroprevalence meta-analysis. Double arcsine transformed proportion of individual studies is plotted against the sample size. The symmetrical distribution of studies in the funnel plot suggests an absence of publication bias

No publication bias was observed through the graphical assessment of the funnel plot (Fig. **5**) and the Egger test for funnel plot asymmetry (p-value = 0.23).

## Discussion

We evaluated the seroprevalence of HCV infection in Burkina Faso between 1990 and 2020 in several settings and populations. The reported seroprevalence in low-risk groups was 3.72% (95% CI: 3.20–4.28) and may reflect that of the general population, classifying the country among those with intermediate seroprevalence [8]. Previous analyses reported a seroprevalence between 4.9 and 6.1% in Burkina Faso, ranking the country among those with the highest seroprevalence in West Africa [10, 12]. However, these reports included high-risk populations in their estimations. In addition, as HCV seroprevalence decreased over time, the inclusion of more recent studies in our review may explain our lower rate. The rate reported in this review is similar to that of the only nationwide survey conducted in 2010, which found a weighted seroprevalence of 3.6% (95% CI: 3.3–3.8) in the general population [9].

Although non-significant, a decreasing trend was observed over the past three decades and can be attributed to several factors, including the improvement of transfusion and injection safety. Since 2000, blood transfusion in Burkina Faso is managed by the national center for blood transfusion (CNTS), which has the capacity for infection screening. However, in 2009, its production capacity covered only half of the needs, with the remaining directly collected in the health facilities not supplied by the CNTS. A survey of 42 of these health centers showed that 14.3% were not routinely performing HCV testing [23]. In addition, a residual risk of HCV transmission persists, estimated at one in 213 donations, as blood screening for HCV is only based on the detection of anti-HCV antibodies [24, 25]. Therefore, nationwide coverage of the blood supply by the CNTS and nucleic acid testing for HCV is recommended to reduce the risk of transfusion-transmitted hepatitis C.

Improved injection safety is the other factor that could explain the downward trend of infection rates. Indeed, in 1996, it was estimated that 11% of rural and 80% of urban health centers were using new and sterile syringes and needles for injections [26]. In 2000, a survey of 52 nationally representative health facilities found that this proportion increased to 96%, and no shortage of syringes or needles was reported in those health centers [27].

The pooled seroprevalence of HCV infection among pregnant women indicates a risk for MTCT, estimated at 4.2–7.8% among viremic women [28]. Nevertheless, the WHO does not recommend routine testing of pregnant women for HCV infection, as currently there is no treatment to prevent MTCT, and DAAs are not indicated during pregnancy [2]. Therefore, adequate detection and treatment of childbearing age women during the preconception period could be recommended.

Rural communities were understudied, although they accounted for 73.7% of the latest national population census [15]. Therefore, the reported seroprevalence may underestimate the magnitude of the actual situation in rural settings. It is thus essential to assess the burden of HCV infection on rural populations, as limited care access, low literacy, and low socioeconomic status are known factors of HCV infection [4, 29].

Few studies were conducted among key populations for HCV infection. Interestingly, two studies included MSM and one involved sex workers, two hard-to-reach groups. However, no study was conducted among PWID. Evidence exists about injecting drug usage in Burkina Faso, but no data are available on PWID numbers [30]. Monitoring the extent of injecting drug use and HCV transmission among PWID and other key populations (e.g., MSM and sex workers) should be implemented.

The pooled HCV RNA prevalence among low-risk populations was 1.65% (95% CI: 0.74–2.89%) and may be indicative of the prevalence in the general population. By applying this rate to the total population of the country in 2019 [15], approximately 301,174 people are estimated to be active HCV carriers in Burkina Faso. The Polaris observatory estimated the total number of active carriers in Burkina Faso at 247,000 in 2015, corresponding to a viremic prevalence of 1.3% (95% CI: 1.0–1.4%), and higher than the global prevalence of 1% [31]. As HCV infection can be treated with highly effective DAAs, public effort should be undertaken to identify active carriers and link them to care.

Only six studies evaluated HCV genotype distribution, and genotypes 2 and 1 were the most prevalent, as reported by previous publications [4, 12, 32]. Since the advent of pangenotypic DAAs, genotype determination is considered of little interest in deciding the adequate treatment for chronic HCV [2, 6]. However, recent reports of treatment failures with pangenotypic DAAs in patients of African descent infected with genotypes 1 and 4 [33, 34] are alarms for monitoring circulating genotypes and evaluating the effectiveness of HCV treatment.

Our study is limited by the small sample size in rural and high-risk populations and the diversity of anti-HCV antibody measurement methods. Also, as expected from a meta-analysis of prevalence data, significant heterogeneity was observed and could be attributed to the various populations and geographic areas [13]. These factors may have underestimated or overestimated the actual seroprevalence. Despite these limitations, our results are valuable in guiding public health response in a setting where data on HCV infection in specific populations and settings are scarce.

## Conclusions

In conclusion, the seroprevalence of HCV infection is intermediate in Burkina Faso, with a decreasing trend over the past 30 years, due to improved blood transfusion and injection safety. There is a paucity of data at the national level and for rural and high-risk populations. The fight against hepatitis C infection requires high-quality and nationally representative data to guide public health response. Although general population testing is recommended, special attention should be paid to rural and high-risk populations, with screening and linkage to care.

## Supplementary Information

**Additional file 1: Table S1.** (Database search). **Table S2.** (Quality appraisal of included studies using the Joanna Briggs Institute checklist for prevalence studies).

## Data Availability

The dataset used and analyzed in this study is available from the corresponding author on reasonable request.

## Abbreviations

HCV: Hepatitis C virus
DAA: Direct-acting antivirus
LMIC: Low- and middle-income countries
RNA: Ribonucleic acid
PWID: Persons who inject drugs
MTCT: Mother-to-child transmission
HCC: Hepatocellular carcinoma
HIV: Human immunodeficiency virus
MSM: Men having sex with men
ELISA: Enzyme-linked immunosorbent assay
CLEIA: Chemiluminescence enzyme immunoassay
CNTS: National center for blood transfusion
CI: Confidence Interval
