# Supplementary material for: Epidemiologic profile of hepatitis C virus infection and genotype distribution in Burkina Faso: a systematic review with meta-analysis
Source: BMC Infect Dis. 2021 Nov 1;21:1126. doi: 10.1186/s12879-021-06817-x (PMC8561994; doi:10.1186/s12879-021-06817-x)
Supplement: Supplementary file 1 — Additional file 1: Table S1. (Database search). Table S2. (Quality appraisal of included studies using the Joanna Briggs Institute checklist for prevalence studies). [file 12879_2021_6817_MOESM1_ESM.docx]

**Table S1: Database search**

| **No** | **Database** | **Search query** | **Number of results** |
| --- | --- | --- | --- |
| **1** | **PubMed** | ("hepatitis C" OR HCV OR “Hep C”) AND (prevalence OR seroprevalence) AND "Burkina Faso" | 32 |
| **2** | **Web of Science** | (TS=HCV OR TS=Hepatitis c OR TS=hep C) AND (TS=prevalence OR TS=seroprevalence) AND TS=Burkina Faso | 43 |
| **3** | **Scopus** | (ALL (hepatitis AND C) OR ALL (HCV) OR ALL (hep AND c) AND ALL (seroprevalence) OR ALL (prevalence) AND TITLE-ABS-KEY (Burkina AND Faso)) | 137 |
| **4** | **African Index Medicus** | "hepatitis" "HCV" "hepatite" "burkina" | 165 |

**Table S2: Quality appraisal of included studies using the Joanna Briggs Institute checklist for prevalence studies**

| **Author, Year of publication** | **Q1. Was the sample frame appropriate to address the target population?** | **Q2. Were study participants sampled in an appropriate way?** | **Q3. Was the sample size adequate?** | **Q4. Were the study subjects and the setting described in detail?** | **Q5. Was the data analysis conducted with sufficient coverage of the identified sample?** | **Q6. Were valid methods used for the identification of the condition?** | **Q7. Was the condition measured in a standard, reliable way for all participants?** | **Q8. Was there appropriate statistical analysis?** | **Q9. Was the response rate adequate, and if not, was the low response rate managed appropriately?** |
| --- | --- | --- | --- | --- | --- | --- | --- | --- | --- |
| Jeannel, 1998a | Yes | No | No | Yes | Yes | Yes | Yes | Yes | Yes |
| Jeannel, 1998b | No | Yes | Yes | No | Yes | Yes | Yes | Yes | Yes |
| Ilboudo, 2003 | Yes | Unclear | No | Yes | Yes | Yes | Yes | Yes | Yes |
| Simpore, 2005 | Yes | Yes | Yes | Yes | Yes | Yes | Yes | Yes | Yes |
| Serme, 2006 | Yes | Unclear | No | Yes | Yes | Yes | Yes | Yes | Yes |
| Kania, 2009 | Yes | Yes | Yes | No | Yes | Yes | Yes | Yes | Yes |
| Collenberg, 2006a | Yes | Unclear | No | Yes | Yes | Yes | Yes | Yes | Yes |
| Collenberg, 2006c | Yes | Yes | No | Yes | Yes | Yes | Yes | Yes | Yes |
| Collenberg, 2006b | Yes | Unclear | No | Yes | Yes | Yes | Yes | Yes | Yes |
| Collenberg, 2006d | Unclear | Unclear | No | Yes | Yes | Yes | Yes | Yes | Yes |
| Simpore, 2006 | No | Unclear | No | Yes | Yes | Yes | Yes | Yes | Yes |
| Ouedraogo A, 2012 | Yes | Unclear | No | Yes | Yes | Unclear | Unclear | Yes | Yes |
| Nagalo, 2011 | Yes | Unclear | Yes | Yes | Yes | Yes | Yes | Yes | Yes |
| Nagalo, 2012 | Yes | Unclear | Yes | Yes | Yes | Yes | Yes | Yes | Yes |
| Zeba, 2011 | No | Unclear | Yes | Yes | Yes | Yes | Yes | Yes | Yes |
| Kirakoya-Samadoulougou, 2014 | Yes | Unclear | Yes | No | Yes | Yes | Yes | Yes | Yes |
| Meda, 2018a | Yes | Yes | Yes | Yes | Yes | Yes | Yes | Yes | Yes |
| Meda, 2018b | Yes | Yes | Yes | Yes | Yes | Yes | Yes | Yes | Yes |
| Kania, 2013 | No | No | No | Yes | Yes | Yes | Yes | Yes | Yes |
| Zeba, 2014 | Yes | Unclear | Yes | Yes | Yes | Yes | Yes | Yes | Yes |
| Tao, 2013 | Yes | Unclear | Yes | Yes | Yes | Yes | Yes | Yes | Yes |
| Zeba, 2012 | No | Unclear | Yes | Yes | Yes | Yes | Yes | Yes | Yes |
| Kissou, 2017 | No | Unclear | No | Yes | Yes | Yes | Yes | Yes | No |
| Yooda, 2019 | Yes | Yes | Yes | Yes | Yes | Yes | Yes | Yes | Yes |
| Diarra, 2017 | No | No | No | Yes | No | Yes | Yes | Yes | Yes |
| Yooda, 2018 | Yes | Unclear | Yes | Yes | Yes | Yes | Yes | Yes | Yes |
| Lingani, 2020a | Yes | Yes | No | Yes | Yes | Yes | Yes | Yes | Yes |
| Lingani, 2020b | Yes | Yes | No | Yes | Yes | Yes | Yes | Yes | Yes |
| Tao, 2014 | No | No | Yes | Yes | Yes | Yes | Yes | Yes | Yes |
| Simpore, 2004 | No | Unclear | Yes | Yes | Yes | Yes | Yes | Yes | Yes |
| Ouedraogo A, 2018 | No | No | Yes | Yes | Yes | Yes | Yes | Yes | Yes |
| Pietra, 2008 | Yes | No | No | Yes | Yes | Yes | Yes | Yes | Yes |
| Ekouevi, 2018 | Unclear | No | No | No | Yes | Yes | Yes | Yes | Yes |
| Sawadogo, 2015 | Yes | Unclear | No | Yes | Yes | Unclear | Unclear | Yes | Yes |
| Ouedraogo HG, 2018 | Yes | No | No | Yes | Yes | Yes | Yes | Yes | Yes |
| Ouedraogo HG, 2019 | Yes | No | No | Yes | Yes | Yes | Yes | Yes | Yes |
| Dah, 2019 | No | No | No | Yes | Yes | Unclear | Yes | Yes | Yes |
